# Muscle power amplification may enhance sprint performance and broaden the thermal performance of the lizard *Sceloporus occidentalis*. I: Sprint performance and joint powers

**DOI:** 10.1242/jeb.252628

**Published:** 2026-10-23

**Authors:** Jordan Balaban, Natalie C. Holt, Anthony Cobos, Emanuel Azizi

**Affiliations:** ^1^ Department of Biology, Barnard College, Columbia University, 3009 Broadway, New York, NY 10027, USA; ^2^ Department of Evolution, Ecology and Organismal Biology, University of California, Riverside, 900 University Ave, Riverside, CA 92521, USA; ^3^ Department of Ecology and Evolutionary Biology, University of California, Irvine, Irvine, CA 92697, USA

**Keywords:** Elastic strain energy, Muscle–tendon unit, Temperature, Fence lizard

## Abstract

The maintenance of locomotor performance across temperatures is critical to ectotherm fitness but is limited by the thermal sensitivity of the myosin ATPase enzyme. However, sprint performance in lizards appears more thermally robust than the muscles powering it, potentially due to power amplification by elastic tendons. To explore this, we determined the thermal sensitivity of *Sceloporus occidentalis* acceleration at 15–35°C, used inverse dynamics to estimate *in vivo* muscle powers at hindlimb joints and compared these to predicted maximum muscle powers. The *Q*_10_ of acceleration was 1.7–2.5, compared with literature *Q*_10_ values of 2–3 for muscle power. Maximum *in vivo* power was ∼180, 622 and 881 W kg^−1^ at the ankle, ∼127, 393 and 433 W kg^−1^ at the knee, and ∼41, 176 and 193 W kg^−1^ at the hip at 15°C, 25°C and 35°C, respectively. Comparison with predicted maximum muscle powers suggest amplifications of ∼2.2, 3.8 and 2.7-fold at 15°C, 25°C and 35°C at the ankle. There was little power amplification at the knee and hip except for a ∼2.4-fold amplification at the knee at 25°C; hip power appeared sub-maximal at 35°C. This is consistent with the limited presence of in-series elastic elements at the knee and, particularly, the hip. These results suggest that power amplification at the ankle is critical to the sprint performance of *S. occidentalis* and its thermal robustness of 25–35°C, but that there is an additional limit to sprint performance at 35°C that may contribute to the apparent thermal robustness.

## Teaser

**Summary:** Power amplification at the ankle may be critical to the sprint performance of *Sceloporus occidentalis*, and its thermal robustness, but there is an additional limit to sprint performance at high temperatures.

## INTRODUCTION

To survive and reproduce, animals must move quickly enough to avoid predation and capture prey. Since ectotherms rely on environmental sources of heat and do not always maintain constant body temperatures despite behavioral thermoregulation (Christian et al., 1983), they have the additional challenge of maintaining performance across varying body temperatures, especially if their predators are homeotherms. The effects of temperature on locomotor performance are driven by the thermal sensitivity of the myosin ATPase enzyme kinetics responsible for producing the muscle force and work required for movement (Huey and Kingsolver, 1989). This effect of temperature on muscle performance results in the observed changes to sprint performance, jump distance, swimming speed and even predator–prey dynamics across a broad range of ectothermic species (Allan et al., 2015; Bennett, 1990; Herrel et al., 2007; Hirano and Rome, 1984; Huey and Stevenson, 1979; Navas et al., 1999; Peplowski and Marsh, 1997). However, the thermal sensitivity of organismal performance on which selection acts (Arnold, 1983) is often less than the thermal sensitivity of the performance of muscles powering these movements (Anderson and Deban, 2010; Anderson et al., 2014; Deban and Lappin, 2011; Else and Bennett, 1987; Marsh and Bennett, 1985, 1986a,b; Peplowski and Marsh, 1997; Whitford et al., 2020).

The thermal sensitivity of muscle and organismal performance is described by the temperature coefficient, *Q*_10_, the change in performance with a 10°C change in temperature. The *Q*_10_ of muscle maximum muscle shortening velocity is typically ∼2. However, *Q*_10_ values as low as 1.6 for frog jumping (Hirano and Rome, 1984) and 1.1 for chameleon tongue projection (Anderson and Deban, 2010) have been reported. There are a variety of reasons why the thermal sensitivity of organismal performance may appear to be less than the thermal sensitivity of the muscle powering this performance. Warm blood may be shunted to important muscles such that their temperature is higher than body temperature thereby enabling higher performance, as in swimming tuna (Altringham and Block, 1997). Alternatively, muscle performance may not be maximal at high temperatures, such that organismal performance may be maintained at low temperatures by increasing muscle recruitment, as observed in the predatory strikes of rattlesnakes (Whitford et al., 2020). Finally, organisms may circumvent the constraints of reduced temperature by utilizing thermally independent tissues, such as elastic connective tissues, to actuate movement and retain performance across a broad range of temperatures (Anderson and Deban, 2010; Kubo et al., 2005; Peplowski and Marsh, 1997; Rigby et al., 1959).

In vertebrates, muscle fibers often operate in series with spring-like tendons, aponeuroses, and other elastic connective tissues in a muscle–tendon unit (MTU). These series elastic elements can temporally decouple muscle length changes from movement by storing and releasing elastic energy. Muscle fibers shorten slowly and some form of latching prevents this muscle shortening from causing movement of the joint, instead directing this muscle work into elastic strain energy in series elastic elements. Release of this latching allows recoil of these series elastic elements and imparts this stored energy on the body at a much faster rate than muscle could alone (Abbott et al., 2019; Astley and Roberts, 2014; Azizi and Roberts, 2010; Divi et al., 2020; Holt and Mayfield, 2023; Ilton et al., 2018; Longo et al., 2019; Mendoza et al., 2023a; Roberts and Azizi, 2011). The total work done by muscle does not change, but the recoil of series elastic elements allows for a much faster release of the muscle work done. Historically, this has been referred to as power amplification, reflecting the premise that the force–velocity relationship of muscle limits muscle power, but that this constraint can be overcome by the recoil of elastic tissues (Bennet-Clark, 1975; Aerts, 1998; Roberts and Marsh, 2003; Roberts and Azizi, 2011). More recent work suggests that this process may be more accurately described by elastic recoil increasing the kinetic energy capacity of the muscle and allowing the muscle to deliver more of its maximum work capacity over the available time and displacement (Labonte, 2023; Labonte and Holt, 2024). However, for now, we retain the terminology of power amplification to reflect the methods used, both historically and in this study, to explore this phenomenon.

Unlike muscles, whose dependence on enzymatic processes give rise to strong thermal sensitivities, series elastic elements are passive mechanical structures that are largely temperature insensitive across biologically relevant temperatures (Kubo et al., 2005; Rigby et al., 1959). Therefore, series elastic elements can enhance organismal performance across a thermal range by mitigating the effects of low temperature on muscle shortening velocity. This phenomenon has been documented across a wide range of vertebrates, and a variety of activities, such as tongue projection in chameleons, salamanders and toads (Anderson and Deban, 2010; Lappin et al., 2006; Scales et al., 2016), and jumping in frogs, bushbabies and humans (Aerts, 1998; Astley and Roberts, 2014; Kurokawa et al., 2003; Longo et al., 2019; Peplowski and Marsh, 1997), and has been demonstrated to confer not only enhanced performance, but also increased thermal robustness in ectotherms (Anderson and Deban, 2010; Deban and Lappin, 2011; Lappin et al., 2006; Peplowski and Marsh, 1997; Scales et al., 2016).

The relatively lower degree of thermal sensitivity of organismal performance compared with muscle performance has also been documented in the western fence lizard *Sceloporus occidentalis*. The sprint speed of these lizards has a *Q*_10_ of around 1.2 between 25°C and 35°C (Marsh and Bennett, 1986b), despite the maximum shortening velocity and power output of the iliofibularis muscle of this species showing a typical *Q*_10_ of ∼2 across physiologically relevant temperatures from 15°C to 35°C (Marsh and Bennett, 1986a). This low thermal sensitivity of sprint performance may be due to maximum muscle capacity not being required above 25°C and a different constraint to speed and acceleration, so limiting performance at high temperatures. However, in two other lizard species, including another *Sceloporus* species, it has been demonstrated that there is only just sufficient muscle power to drive acceleration at ∼35°C (Curtin et al., 2005; McElroy and McBrayer, 2010). Consequently, it seems unlikely that these lizards could increase muscle recruitment to account for the effects of lower temperature. Instead, passive, elastic structures may amplify muscle power to maintain sprint performance across temperatures (Marsh and Bennett, 1986a,b). However, power amplification during sprinting in this species would be surprising, both because of the cyclical nature of the activity and because small running or hopping species have been suggested to be unable to effectively store and return elastic energy (Biewener and Blickhan, 1988; Biewener et al., 1981; Pollock and Shadwick, 1994).

Power amplification has largely been demonstrated in one-shot, ballistic behaviors. Such movements typically provide opportunity for a long preparatory phase in which muscle can shorten slowly and store significant elastic strain energy often before any observable movements. The first step of an acceleration can be considered ballistic, but unlike the saltatory jumping of a frog or the tongue projection of a chameleon, an acceleratory step must set the animal up for the cyclical contractions occurring during subsequent steps. The high-frequency cyclical movement of the limbs required for running presumably limits this preparatory phase and requires a high degree of coordination which may limit the capacity to store energy in series elastic elements and amplify power. However, power enhancement or amplification (Richards and Sawicki, 2012) has been demonstrated at push-off during human walking (Fukunaga et al., 2001; Hof et al., 1983; Lichtwark and Wilson, 2006), at the onset of swing in galloping horses (Wilson et al., 2003) and in the second half of stance in accelerating turkeys (Roberts and Scales, 2002) and humans (Lai et al., 2016; Lin and Pandy, 2022; Miller et al., 2012).

Some studies have argued that, owing to the relatively large diameter of tendon compared with muscle, small animals are not capable of storing sufficient elastic strain energy in their tendons to effectively use recoil to enhance cyclical movements such as running or hopping (Biewener and Blickhan, 1988; Biewener et al., 1981; Pollock and Shadwick, 1994). However, other work suggests that the tendons of small animals can still store a significant proportion of elastic strain energy (Bullimore and Burn, 2005; Christensen et al., 2022), and simulation studies suggest that the long toe tendons of the relatively small zebra-tailed lizards can store ∼40% of stride work under biologically relevant loads (Li et al., 2012). Moreover, it has been suggested that power amplification requires a stiffer tendon than the cycling of elastic energy for metabolic efficiency, which is presumed not to be possible in these small running and hopping species (Holt and Mayfield, 2023), thus potentially making higher stiffness advantageous.

Here, we determine the acceleration that can be achieved across a range of body temperature in the western fence lizards, use an inverse dynamics approach to determine instantaneous joint powers during these accelerations and compare these joint powers to predicted muscle powers across temperatures based on previously measured values (Balaban and Azizi, 2017) and literature estimates of *Q*_10_ (Anderson and Deban, 2010; Marsh and Bennett, 1986a,b). Such an approach is commonly used to measure joint powers in vertebrates and has been used to provide evidence for power amplification (Astley and Roberts, 2014; Carroll et al., 2008; Farris and Sawicki, 2012). If our calculated joint powers exceed the predicted maximal muscle power available, then series-elastic elements are implicated in cycling elastic strain energy to amplify muscle power (Astley and Roberts, 2014; Carroll et al., 2008; Farris and Sawicki, 2012). If joint powers and acceleration performance exhibit lower thermal sensitivity than predicted muscle powers, then this power amplification is implicated in increasing thermal robustness (Anderson and Deban, 2010; Deban and Lappin, 2011; Lappin et al., 2006; Peplowski and Marsh, 1997; Scales et al., 2016).

## MATERIALS AND METHODS

In this study, we combined an inverse dynamics analysis with morphological data to estimate maximal instantaneous *in vivo* muscle power. First, we estimated instantaneous joint powers about the ankle, knee, and hip joints during the first step of acceleration at 15, 25 and 35°C using three-dimensional kinematics and ground reaction force data. We then used diffusible iodine contrast-enhanced computed tomography (DICE-CT) to measure muscle moment arms and estimate maximum instantaneous *in vivo* muscle power output at each of these joints. We then compared the apparent *in vivo* muscle power output predicted by inverse dynamics across temperature with the maximum *in vitro* muscle power based on literature values (Balaban and Azizi, 2017; Marsh and Bennett, 1986a,b). If estimates of *in vivo* muscle power exceed those reported for muscle in the literature at the same temperature, then we presume that these apparent *in vivo* muscle powers do not reflect muscle alone, but also reflect contributions from elastic tissues. *In vitro* estimates of muscle power probably overestimate what is possible *in vivo*, especially during cyclical contractions, as used here. Hence, this comparison is likely to represent an underestimation of the degree of power amplification.

### Animals

Nine western fence lizards, *Sceloporus occidentalis* Baird and Girard 1852 (9.18*±*2.06 g, mean*±*s.d.), were caught on the University of California, Irvine campus using a California Department of Fish and Wildlife scientific collector's permit SC-12906 issued to J.P.B. Lizards were housed individually or in pairs with a 12 h:12 h light:dark cycle with UV broad-spectrum light (Exo Terra Repti Glo 2.0, 24) and a heating bulb (Exo Terra Sun Glo Basking Spot Lamp, 75 W) placed above one side of the cage to allow for behavioral thermoregulation. They were housed in terraria measuring ∼50×30×30 cm with a sandy substrate, given water *ad libitum* and fed crickets supplemented with calcium to satiation twice per week. This work was carried out at UC Irvine under Institutional Animal Care and Use Committee protocol no. 2013-3110.

### Acceleration kinematics

We acclimated the lizards for at least 2 h in an environmental chamber held at 15°C (*N*=4), 25°C (*N*=7), or 35°C (*N*=9). We measured cloacal temperature prior to any testing to ensure body temperature matched the environmental temperature. After acclimation, lizards were placed on a custom track in which we embedded a force transducer (nano17; ATI Industrial Automation, Apex, NC, USA). All running surfaces were covered with 80-grit sandpaper to provide traction. We marked the left hindlimb joints as well as the center of mass (COM) of the animals with white-out and a black marker at the center for digitizing (Fig. 1A). We placed the lizards on the track so that their left hindfoot, but no other body part, was fully in contact with the force transducer. We then encouraged them to accelerate toward a darkened chamber while filming at 1000 Hz with two Edgertronic high-speed cameras (Model SC1, Sanstreak Corporation, San Jose, CA, USA) placed dorsally and laterally to the force plate. A custom-built 48-point calibration object was used to calibrate the two camera views. Force data were collected at 1000 Hz using a 16-bit data acquisition system (National Instruments, TX, USA) and initially analyzed using Igor Pro software (V 6.22A, Wavemetrics, Lake Oswego, OR, USA) before being imported into MATLAB (MathWorks, Natick, MA, USA) for further analysis. Cameras were triggered using an external trigger. The trigger signal was split and simultaneously sent to the cameras and to Igor Pro to synchronize the force and kinematic data.

**Fig. 1.**
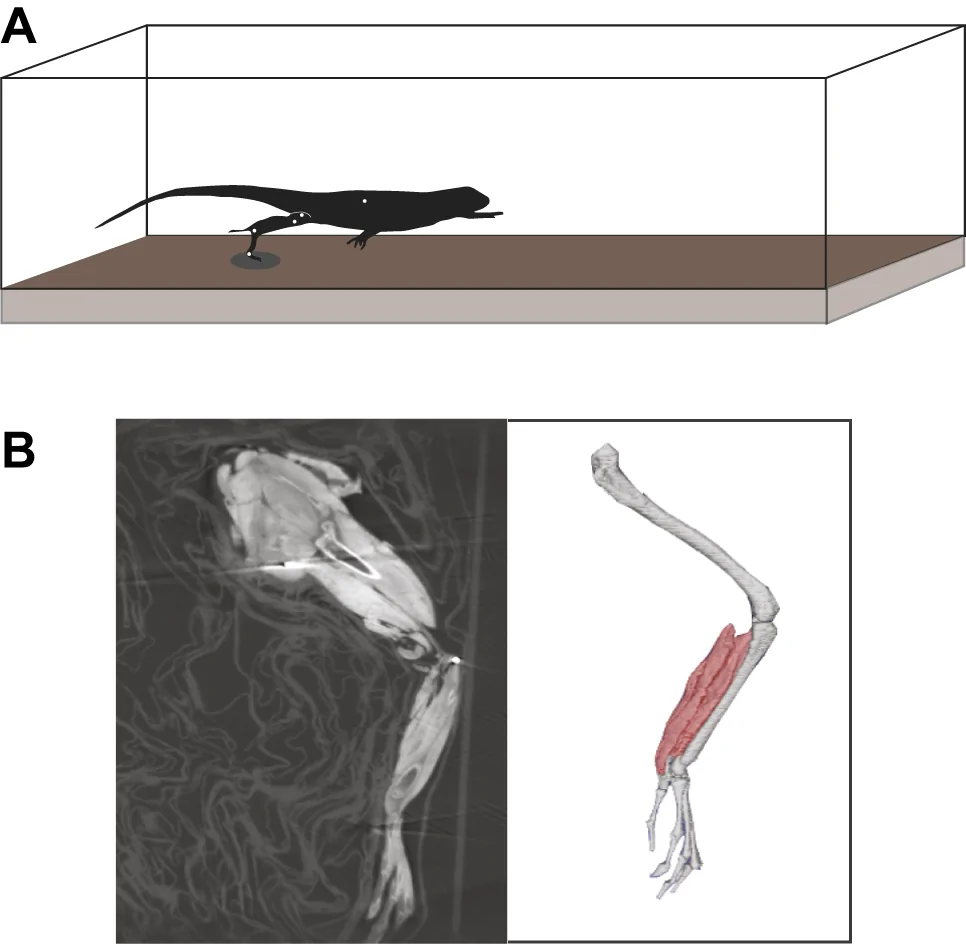
**Experimental setup to measure sprint performance in fence lizards.** (A) Lizards were marked with whiteout and sharpie at the toes, ankle, knee, hip and center of mass. They were then filmed with two high speed cameras as they accelerated off a six-axis force transducer (gray circle) to measure the kinematics and ground reaction forces in three dimensions. (B) Sample output from DICE-CT scan. On the left is a single CT slice. Musculature is stained with iodine. On the right is a reconstruction of the hindlimb bones and ankle extensor muscles. Muscle in-lever distances were taken from these reconstructions.

We used DLT software in MATLAB (Hedrick, 2008) to digitize the 3D motion of the joint centers and the lizard centers of mass. To orient the camera frame of reference to the force plate frame of reference, we digitized four points on the surface of the force transducer corresponding with the positive and negative *x* and *y* force vectors, while calculating the *z* force vector as orthogonal to *x* and *y*. Coordinate and force data were low-pass filtered at 50 Hz using a second-order Butterworth filter applied bidirectionally prior to further analysis. Animal acceleration values were calculated from the second derivative, with respect to time, of the center of mass (COM) marker.

### Inverse dynamics analysis

We wrote custom MATLAB code using Denavit–Hartenberg parameters (Craig, 2005) to analyze the kinematics and run an inverse dynamics analysis (Fig. 2). The center of pressure of the foot was estimated as the *x* and *y* position of the metatarsophalangeal joint of toe 3, along with the *z* position of the floor. Given that we do not know the relative force on each of the toes in contact with the ground, this is a conservative estimate of the position of the center of pressure and, if anything, leads to an underestimate of joint powers.

**Fig. 2.**
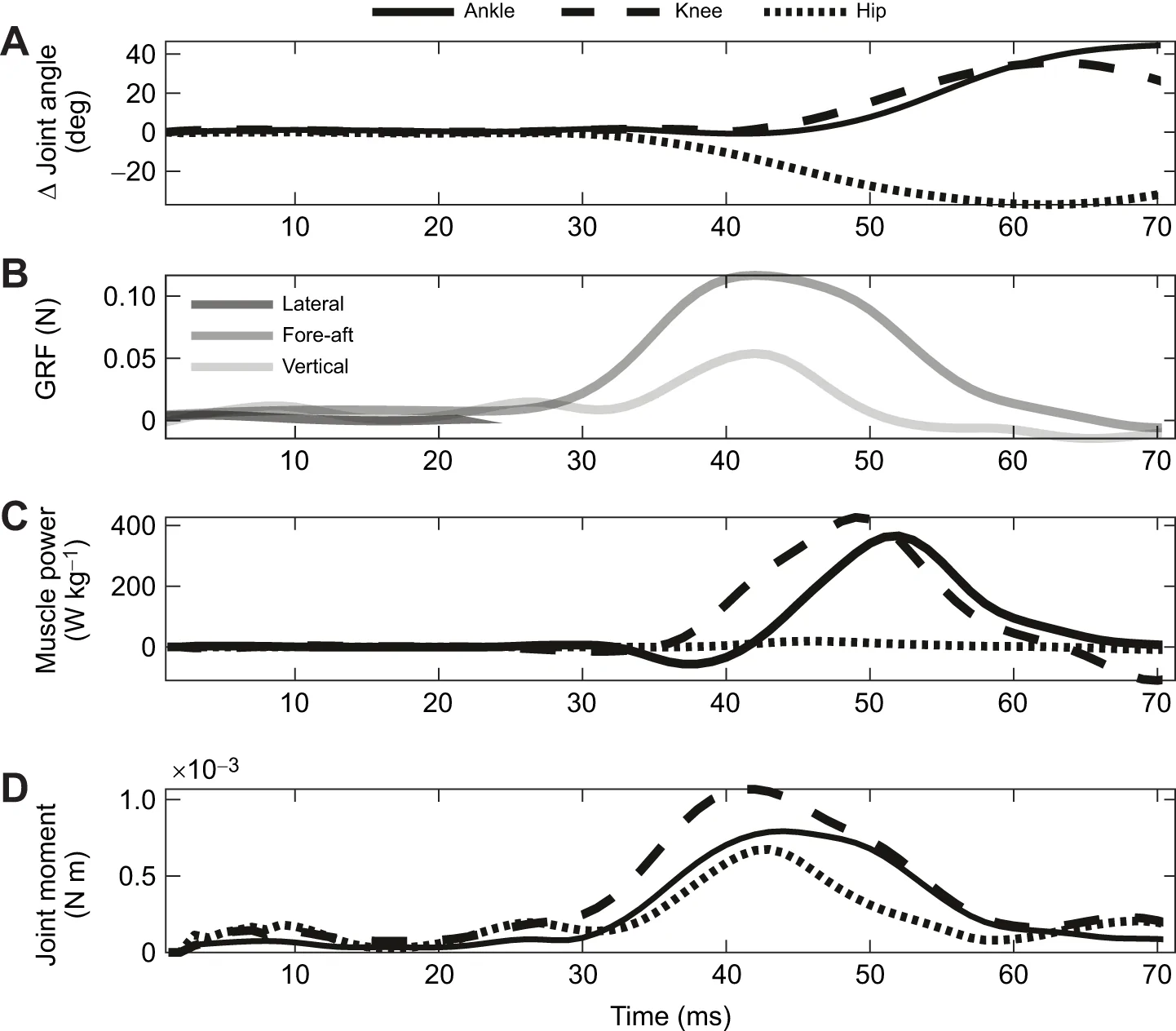
**Joint angle, total ground reaction force, mass-specific muscle power and joint moments for a single step in the fence lizard *Sceloporus occidentalis*.** (A–D) Example output from one step for the ankle, knee and hip values showing (A) joint angle (angle at rest set to 0), (B) total ground reaction force (GRF), (C) mass-specific muscle power and (D) joint moments calculated from custom MATLAB code used to analyze kinematics and run inverse dynamics analysis. Time zero is start of acceleration.

We calculated the joint angles using a transformation matrix assuming the knee is a hinge joint. This allowed us to assume that the knee, ankle and hip joints were in the same plane, which we used to calculate the displacement in the foot and center of mass in an orthogonal plane. Angular velocities and angular accelerations were calculated as the first and second derivative, respectively, with respect to time. We then used these joint angles, angular velocities, and accelerations in inverse dynamics calculations to determine net joint moments.

Net joint moments were calculated working distally to proximally from the foot through the ankle, knee and hip using a Newton–Euler inverse-dynamics approach. For each segment we computed *x*, *y* and *z* components of moments as:

(1) $$M_{\mathrm{joint}}=r_{\mathrm{COM}}\times (m\;a_{\mathrm{COM}})+\underset{i}{\sum }\;r_{i}\times F_{i},$$

where r_COM_ is the vector from the joint center to the segment center of mass, *m* is segment mass, **a**_COM_ is the linear acceleration of the segment center of mass, **r***_i_* are position vectors from the joint center to points of force application (the ground reaction force was applied at the estimated center of pressure), and **F***_i_* are external forces including ground reaction and gravitational forces. Segment masses and COM locations were estimated from dissection and DICE-CT data. Joint reaction forces and moments computed for the foot were used as inputs to compute ankle moments; ankle reaction forces and moments were then used to compute knee moments, and so on. Moments were computed in three dimensions and we report the resultant moment:

(2) $$M=\sqrt{M_{x}^{2}+M_{y}^{2}+M_{z}^{2}}.$$

We calculated joint power, **P**_joint_, by multiplying moment, **M**, by angular velocity, ω:

(3) $$P_{\mathrm{joint}}=M\times \omega .$$

Instantaneous mass-specific *in vivo* muscle power, **P**_muscle_, was then calculated by multiplying instantaneous muscle force, **F**_muscle_, by muscle shortening velocity, **V**_muscle_ and dividing by the mass of muscles contributing to joint extension (measured from dissected specimens). A major issue of inverse dynamics is the potential for power transfer between joints to lead to an overestimation of *in vivo* mass-specific muscle power at a given joint. This potential issue is critical here as we compare *in vivo* mass-specific muscle powers at joints to the maximum possible muscle power at that joint from *in vitro* estimates, and interpret *in vivo* powers exceed maximum *in vitro* muscle power at a joint as evidence of power amplification by elastic structures. We take two approaches to address this issue. Firstly, power is likely to be transferred between joints by biarticular muscles. Thus, biarticular muscles were included in the calculation of both joints that they crossed. Secondly, we summed the ankle, knee, and hip joint powers at each time point and divided this by the total muscle mass of the limb to calculate a whole limb maximal *in vivo* mass-specific muscle power. This second approach is very conservative and limits our ability to draw conclusions about specific joints. However, if whole limb *in vivo* mass-specific muscle power exceeds that possible from the total muscle mass (*M*_muscle_), this will provide definitive evidence of power amplification, the location of which can then be clarified by our first approach:

(4) $$P_{\mathrm{muscle}}=\frac{F_{\mathrm{muscle}}\times V_{\mathrm{muscle}}}{M_{\mathrm{muscle}}}.$$

Muscle force was calculated by dividing the net joint moment (**M**) by the estimated in-lever distance (*L*_i_) of the muscle group extending each joint (**F**_muscle_=**M**/*L*_i_). Muscle shortening velocity (**V**_muscle_) was calculated as the derivative of muscle length with respect to time. Muscle length was estimated from joint angles and *L*_i_. All hip extensor muscles were biarticular with the knee and included the femorotibialis, pubo-ischio-tibialis, flexor tibialis externus, and flexor tibialis internus (after Secoy, 1971). The ambiens is a large simple knee extensor muscle, and only crosses the knee joint. The two heads of the gastrocnemius and the smaller popliteus are the ankle extensor muscles, they originate on the tibia and thus are not biarticular (Secoy, 1971 and our observations). Thus, hip mass-specific muscle power was calculated using the hip extensor muscle masses, knee mass-specific muscle power was calculated using knee and hip muscle masses and ankle mass-specific muscle power was calculated using ankle extensor muscle masses.

We calculated moment of inertia (*I*) for each limb segment from the DICE-CT scans (see below) and calculated rotational inertia by multiplying by rotational acceleration (*I*α). However, the limb segment *I* values were low enough to have a negligible effect on joint power and only served to increase the noise of the signal. For this reason, we omitted the *I*α term from the final moment calculations. *Q*_10_ values for acceleration and muscle powers were calculated as the average within individual *Q*_10_ values between 15°C and 25°C (*N*=4) and between 25°C and 35°C (*N*=7).

### DICE-CT

To measure the distance of the skin markers and the muscle insertions to the joint centers we visualized the muscle and bony tissue using diffusible iodine contrast-enhanced computed tomography (DICE-CT) (Gignac et al., 2016) (Fig. 1B). The lizards used in the above acceleration trials were euthanized via an overdose of 5% inhaled isoflurane followed by a double pithing protocol. We then preserved the specimens in 10% formalin solution for at least 3 days. Following this, we isolated the hindlimb and the portion of the trunk with the center of mass markers from the rest of the body. We inserted an insect pin into the marked areas on each joint of the left hindlimb and the center of mass so that the superficial tip of the insect pin was flush with the skin to match skin markers with μCT data. We then removed the skin and placed the samples in 100% Lugol's iodine solution for 5–7 days. Following this, we took μCT scans of the lizards (Xradia VersaXRM 410) with a resolution of 40.231 μm voxel^*−*1^ at the UC Irvine Materials Research Institute (University of California, Irvine, USA). 3D images were rendered using the software program Horos (v.2.4.0) and all structures were assembled and distance measurements taken using MeshLab (v.2016.12) (Cignoni et al., 2008).

### Statistics

All statistical analyses were performed in R (r-project.org). Using the lme4 (https://CRAN.R-project.org/package=lme4; Bates et al., 2015) and car (https://CRAN.R-project.org/package=car) packages, we ran linear mixed effects models using the maximum values for each lizard at each temperature for joint powers, instantaneous *in vivo* muscle powers, joint angular velocities and lizard accelerations. Differences between temperature treatments were determined using a least square means analysis. In all tests, temperature was used as a fixed effect, and individual was used as a random effect to account for individual variation.

## RESULTS

### Acceleration

Maximal instantaneous acceleration (mean±s.e.m.) during the first step was 18.2*±*3.6 m s^−2^ at 15°C (*N*=4), 32.7*±*3.2 m s^−2^ at 25°C (*N*=7) and 52.0*±*3.9 m s^−2^ at 35°C (*N*=9). There was a significant effect of temperature on maximal acceleration (*P<*0.001, LME model with individual as random effect). There was a significant difference between 15°C and 25°C (*P<*0.001, LSM) and between 25°C and 35°C (*P<*0.001, LSM). The *Q*_10_ of maximal acceleration (mean±s.e.m.) was 2.49±0.57 between 15°C and 25°C (*N*=4) and 1.74*±*0.19 between 25°C and 35°C (*N*=7).

### *In vivo* muscle power

The maximum apparent instantaneous *in vivo* ankle muscle power (mean±s.e.m.) in accelerating fence lizards was 180±26 W kg^−1^ (*N*=4) at 15°C, 622±50 W kg^−1^ (*N*=7) at 25°C and 881*±*78 W kg^−1^ (*N*=9) at 35°C. There was a significant effect of temperature on maximum instantaneous *in vivo* ankle muscle power (*P<*0.001, LME model with individual as random effect; Fig. 3). There were significant differences between 15°C and 25°C (*P<*0.001, LSM) and between 25°C and 35°C (*P*=0.001, LSM). The *Q*_10_ of maximum instantaneous *in vivo* ankle muscle power is 3.45*±*0.25 (*N*=4) between 15°C and 25°C, and 1.50*±*0.11 (*N*=7) between 25°C and 35°C. Based on previous *in vitro* measurements of maximum muscle power and its temperature sensitivity in fence lizards, we would predict that muscle should only be able to generate a maximum of 82, 164 and 328 W kg^−1^ at 15°C, 25°C and 35°C, respectively (Balaban and Azizi, 2017). The estimation of instantaneous *in vivo* powers was higher than these expected values by 2.2*±*0.32 fold (*N*=4) at 15°C, 3.79*±*0.30 fold (*N*=7) at 25°C and 2.68*±*0.24 fold (*N*=9) at 35°C at the ankle joint.

**Fig. 3.**
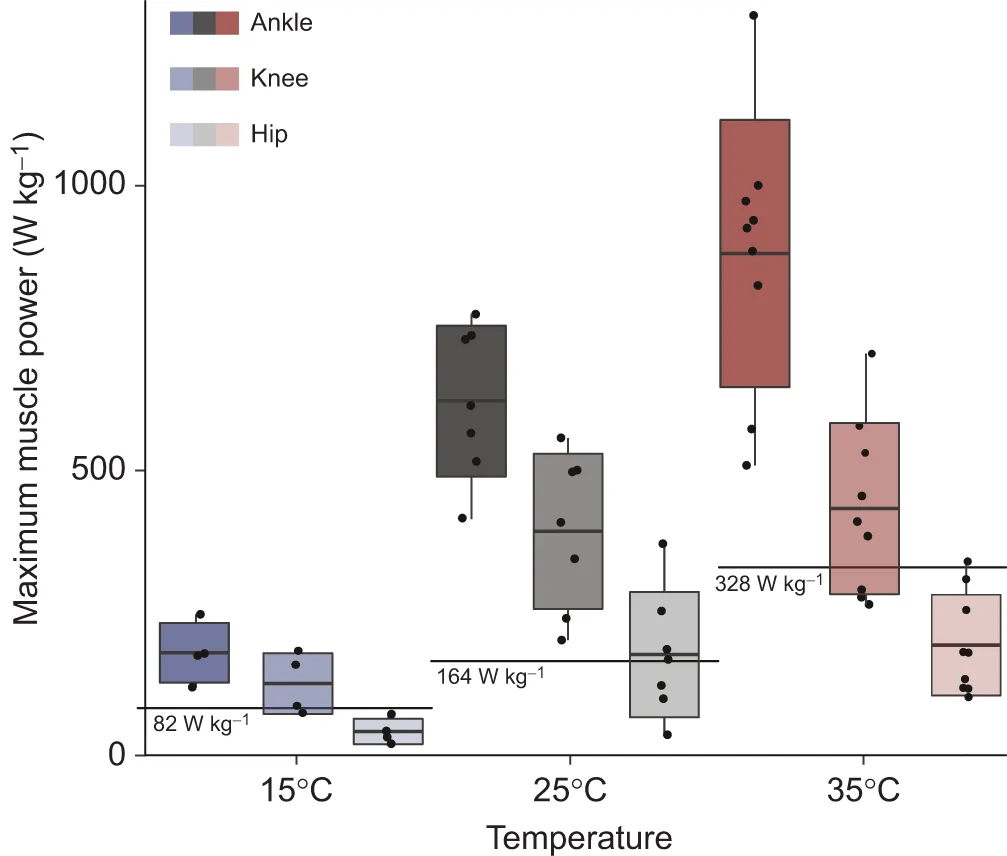
**Maximal ankle, knee and hip muscle power measured in each lizard at 15°C, 25°C and 35°C.** Box plots represent the mean, s.d., minimum and maximum values. Individual data points are plotted with random jitter for visualization. Maximal muscle power at each temperature is estimated from previous work (Balaban and Azizi, 2017) and presented as horizontal line with power values listed next to them. There is a significant effect of temperature on ankle muscle power (*P*<0.001). There is a significant effect of temperature on knee muscle power (*P*<0.001), but there is no difference between 25°C and 35°C (*P*=0.20). Similarly, there is a significant effect of temperature on hip muscle power (*P*<0.001), but there is no difference between 25°C and 35°C (*P*=0.65).

Maximum instantaneous *in vivo* knee muscle power was 127*±*27 W kg^−1^ (*N*=4) at 15°C, 393*±*55 W kg^−1^ (*N*=7) at 25°C and 421*±*52 W kg^−1^ (*N*=9) at 35°C. There was a significant effect of temperature of knee joint power (*P<*0.001, LME; Fig. 3) and significant differences in maximum instantaneous *in vivo* knee muscle powers at 15°C and 25°C (*P<*0.001, LSM), but no differences between 25°C and 35°C (*P*=0.2, LSM; Fig. 3). The *Q*_10_ of instantaneous knee joint power between 15°C and 25°C was 3.20*±*0.45 (*N*=4) and between 25°C and 35°C was 1.32*±*0.19 (*N*=7). Based on previous measurements of muscle power and its temperature sensitivity in fence lizards (Balaban and Azizi, 2017; Marsh and Bennett, 1986a), muscle power was 1.55*±*0.32 (*N*=4) times the expected value at 15°C, 2.39*±*0.31 (*N*=7) at 25°C and 1.31*±*0.15 (*N*=9) at 35°C at the knee joint.

Maximum instantaneous *in vivo* hip muscle power was 40.9*±*11.2 W kg^−1^ (*N*=4) at 15°C, 176*±*42 W kg^−1^ (*N*=7) at 25°C and 193*±*30 W kg^−1^ (*N*=9) at 35°C. There was a significant effect of temperature on maximum instantaneous *in vivo* hip muscle power (*P<*0.001, LME; Fig. 3) and there were significant differences in maximum instantaneous *in vivo* hip joint power between 15°C and 25°C (*P<*0.001, LSM), but not between 25°C and 35°C (*P*=0.65, LSM; Fig. 3). The *Q*_10_ of instantaneous hip muscle power between 15°C and 25°C was 7.18*±*1.98 (*N*=4) and between 25°C and 35°C was 1.66*±*0.61 (*N*=7). Based on previous measurements of muscle power and its temperature sensitivity in fence lizards (Balaban and Azizi, 2017; Marsh and Bennett, 1986a), muscle power was 0.49*±*0.14 times (*N*=4) the expected value at 15°C, 1.07*±*0.25 (*N*=7) at 25°C and 0.58*±*0.09 (*N*=9) at 35°C at the hip joint.

Maximum instantaneous *in vivo* whole limb power was 163*±*29.2 W kg^−1^ (*N* =3) at 15°C, 517±54 W kg^−1^ (*N*=7) at 25°C and 591*±*60 W kg^−1^ (*N*=8) at 35°C. Again, based on previous measurements, muscle power was 1.99*±*0.35 times (*N*=3) greater than the expected value at 15°C, 3.15*±*0.33 (*N*=7) at 25°C and 1.80*±*0.18 (*N*=8) at 35°C (Fig. 4).

**Fig. 4.**
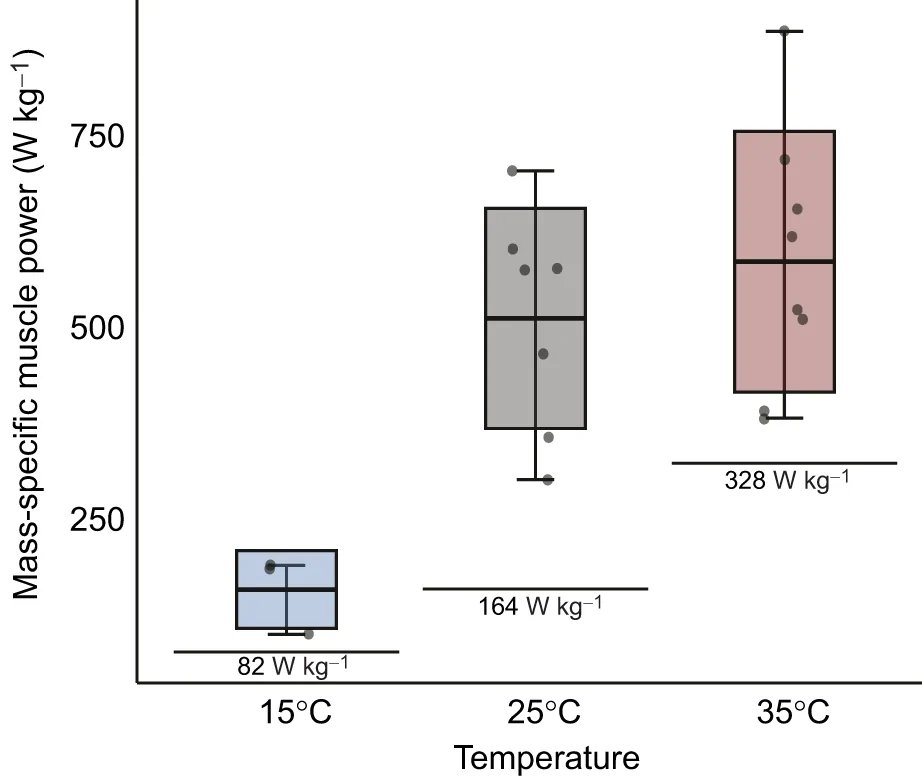
**Maximal instantaneous muscle power summed across all joints measured in each lizard at 15°C, 25°C and 35°C.** Box plots represent the mean, s.d., minimum and maximum values. Individual data points are plotted with random jitter for visualization. Maximal muscle power at each temperature is estimated from previous work (Balaban and Azizi, 2017) and presented as horizontal lines with power values listed.

## DISCUSSION

The goals of this study were to determine whether acceleration and apparent maximum instantaneous *in vivo* muscle powers at joints were greater and showed lower thermal sensitivities than the muscles powering them in the western fence lizard, *S. occidentalis*, and whether power amplification by series elastic elements could explain these effects. We determined maximum acceleration at 15, 25 and 35°C, used inverse dynamics to determine apparent maximum instantaneous *in vivo* muscle powers at all joints and for the whole limb at these temperatures, and predicted muscle powers at these temperatures based on previous *in vitro* data (Balaban and Azizi, 2017; Marsh and Bennett, 1986a). Thermal sensitivities of acceleration and *in vivo* muscle powers at joints were determined as *Q*_10_ values between 15°C and 25°C, and between 25°C and 35°C, and power amplification was determined as the difference between maximum instantaneous *in vivo* muscle powers at joints and predicted maximum muscle powers based on *in vitro* studies at 15, 25 and 35°C, having ruled out the possibility of high maximum instantaneous *in vivo* muscle powers at joints arising from power transfer between joints (Figs 3 and 4). A *Q*_10_ value lower than 2 for acceleration or *in vivo* muscle power indicates a decreased thermal sensitivity and *in vivo* muscle powers that exceed predicted muscle power based on *in vitro* values indicate power amplification by series elastic elements. However, the interpretation of *in vivo* measurements is complicated by the potential for changes in motivation and locomotor strategy across temperatures and contributions from elastic tissues. Hence, *Q*_10_ values must be interpreted in light of the comparison between *in vivo* muscle power and maximum predicted muscle powers.

*Q*_10_ values less than 2 were observed for acceleration, and for ankle, knee and hip muscle power between 25 and 35°C. However, *Q*_10_ values greater than 2 were observed for acceleration, and ankle, knee and hip muscle power between 15 and 25°C, with exceptionally high *Q*_10_ values at the hip (Fig. 3). Power amplification was observed at the ankle and knee at 15, 25 and 35°C, with the greatest degree of amplification occurring at the ankle at 25°C (Fig. 3). However, not only was no power amplification observed at the hip joint, but *in vivo* muscle powers at this joint at 15 and 35°C required only half of the predicted maximum muscle power available at these temperatures, indicating changes in muscle activation due to changing motivation or locomotor strategy (Fig. 3).

The low *Q*_10_ of acceleration (1.74) reported here for the western fence lizard indicates that, as for running which has a *Q*_10_ of 1.2 (Marsh and Bennett, 1986a,b), organismal performance has a lower thermal sensitivity than the muscle tissue powering it. This adds to a body of literature demonstrating this phenomenon across a range of ectothermic species and types of movement (Anderson and Deban, 2010; Deban and Lappin, 2011; Lappin et al., 2006; Marsh and Bennett, 1986a,b; Peplowski and Marsh, 1997; Scales et al., 2016). This maintenance of performance across temperatures may have important fitness consequences for the western fence lizard. The preferred body temperature of these lizards is ∼33°C, and although behavioral thermoregulation can maintain body temperatures close to this optimum for significant periods of the day through the selective use of thermal habitats, thermal models indicate that higher levels of performance at body temperatures of 25°C could increase potentially active times by several hours each day (Buckley et al., 2015; Burgos, 2018).

The combination of low thermal sensitivities of acceleration and *in vivo* muscle powers at joints between 25 and 35°C, and *in vivo* muscle powers that exceed predicted maximum muscle powers at the knee and the ankle joints, particularly at the ankle joint at 25°C, strongly indicate that power amplification by series elastic elements can increase organismal performance and is, at least in part, responsible for the decreased thermal sensitivity of locomotor performance between 25 and 35°C. The high degree of power amplification observed at 25°C may also explain the high temperature sensitivity of ankle and knee joint point power between 15 and 25°C as power amplification at 25°C will increase the performance gap between 15 and 25°C, not because of low thermal performance at 15°C, but rather because of high performance at 25°C.

To amplify muscle power, three elements are required: a contractile element of sufficient force and work capacity, a series elastic element of appropriate stiffness, and a mechanism to control the flow of energy and temporally decouple muscle work from movement (Abbott et al., 2019; Divi et al., 2020; Galantis and Woledge, 2003; Holt and Mayfield, 2023; Ilton et al., 2018; Longo et al., 2019; Mendoza and Azizi, 2021; Roberts and Marsh, 2003). In this system, the extensive musculature of the lizard hindlimb represents the initial source of mechanical energy (Allen et al., 2010; Herrel et al., 2008; Secoy, 1971). There are several elastic elements in the lizard hindlimb that could potentially be used in power amplification (Alexander, 2002; Holt, 2020; Roberts, 2016), such as the extensive aponeurosis of the knee extensors in western fence lizards (our observation) and other lizard species (Herrel et al., 2008), and the tendon and plantar fascia by which the gastrocnemius muscle inserts on to the phalanges in the western fence lizard (our observation) and many other *Sceloporus* species (Secoy, 1971). Indeed, the plantar fascia has been suggested to store and return significant elastic strain energy, for metabolic savings rather than power amplification, in the zebra-tailed lizard (Li et al., 2012).

The degree of power amplification observed here suggests a distinct mechanism enabling the slow flow of muscle work into elastic strain energy followed by the rapid conversion into kinetic energy, rather than simply the effects of inertial loading (Galantis and Woledge, 2003; Roberts and Azizi, 2011; Roberts and Marsh, 2003). It may be that, as has been demonstrated in frog jumping, the increasing mechanical advantage and proximal-to-distal unloading of the limb enables this decoupling by increasing and then decreasing the effective load against which muscle operates (Astley and Roberts, 2014). This pattern of limb actuation has been observed in many running and jumping species including lizards more generally and the western fence lizard examined here (Bels et al., 1992; Aerts, 1998; Astley and Roberts, 2014) (Fig. 5). This hypothesis is supported by the low power amplification at the knee and ankle joint at 15°C being accompanied by a loss of proximal-to-distal limb unloading at this temperature (Fig. 5). Although this mechanism probably cannot achieve the complete decoupling of muscle work from movement seen in activities such as tongue projection and is required for very high levels of power amplification and thermal robustness (Anderson and Deban, 2010), it likely could allow a partial decoupling of muscle work and movement sufficient to explain the relatively modest degree of power amplification and thermal robustness observed here (Peplowski and Marsh, 1997; Abbott et al., 2019; Longo et al., 2019; Mendoza et al., 2023a,b).

**Fig. 5.**
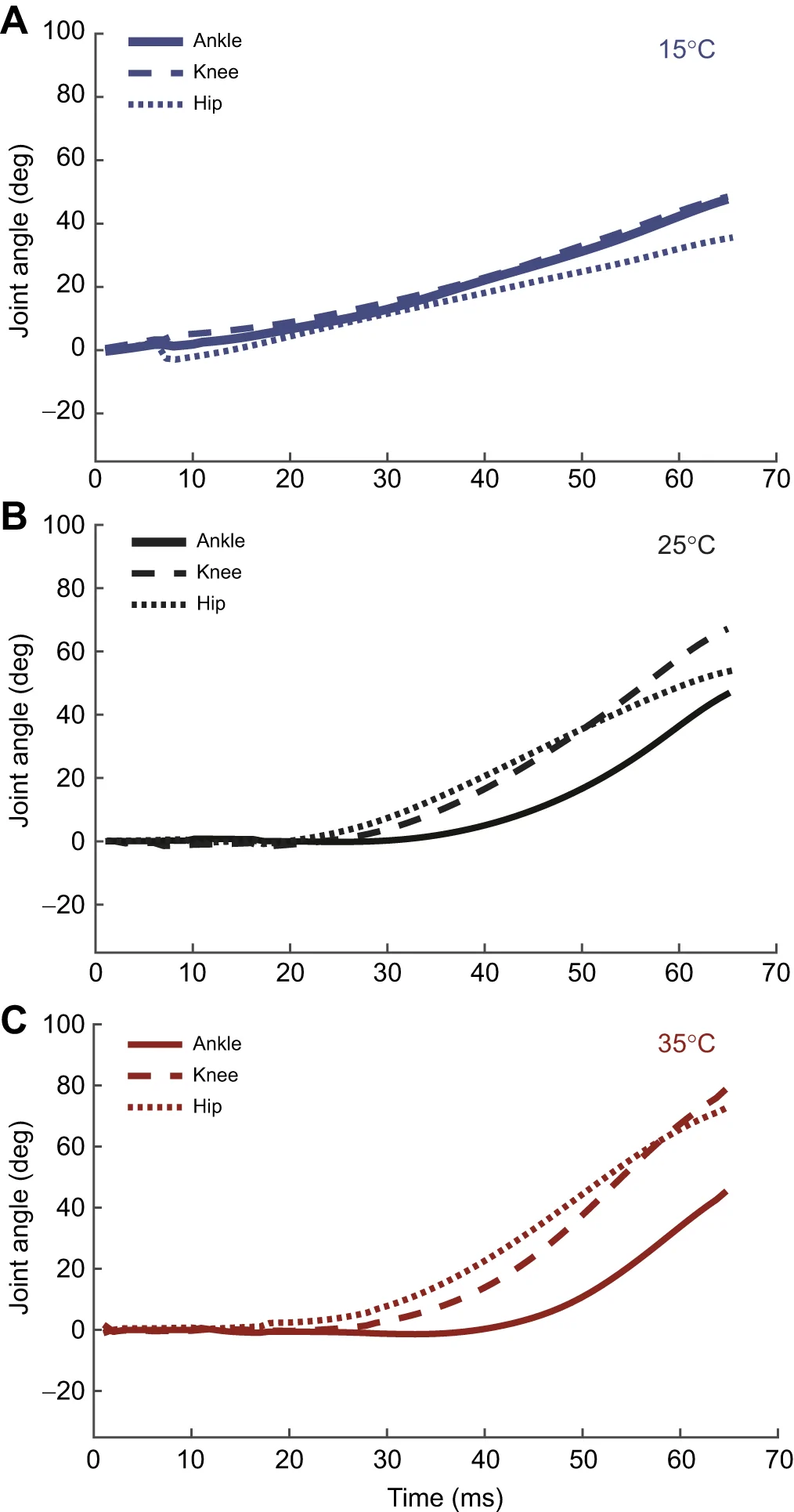
**Average joint angles during acceleration in fence lizards at 15°C, 25°C and 35°C.** (A–C) The kinematics are very similar between 25°C (B) and 35°C (C), but at 15°C (A) there is not the same proximal to distal extension of the limbs (15°C, *N*=4; 25°C, *N*=7; 35°C, *N*=9).

Irrespective of how power amplification is achieved in this system, it is clear that power can be amplified at the ankle and the knee, and that the high degree of power amplification at 25°C contributes to the low thermal sensitivities observed between 25 and 35°C. However, the situation at the hip is a little more complicated and may also contribute to observed *Q*_10_ values. The hip is likely to be entirely muscle driven. Predictions of muscle power are sufficient to explain *in vivo* muscle power at the joint at 25°C, and observed *in vivo* muscle powers are much lower than predicted maximum muscle powers at 15 and 35°C. This lack of power amplification at the hip is consistent with the apparent lack of large-scale series elastic structures at this joint in *Sceloporous* species (our observations; Secoy, 1971). Despite this lack of power amplification, *Q*_10_ values of *in vivo* muscle powers deviate from an expected value of ∼2 at the joint. There appears to be a very high degree of thermal sensitivity between 15 and 25°C, and a very low degree between 25 and 35°C. The high thermal sensitivity between 15 and 25°C is likely driven by *in vivo* muscle powers at this joint which only require 49% of peak predicted muscle power at 15°C but 107% of peak predicted muscle power at 25°C (Fig. 3). Hence, some of the increase in performance with temperature is probably due to a change in muscle activation. It is not clear why this change in muscle activation would occur. However, it may be a reflection of a changing behavioral strategy. At low temperatures, many species of lizards elect to hold their ground and fight rather than flee (Crowley and Pietruszka, 1983; Hertz et al., 1982; Mautz et al., 1992) as the isometric force underlying bite performance has a lower thermal sensitivity than the muscle shortening velocity underlying sprint performance (Herrel et al., 2007). Indeed, during our running trials, lizards at 15°C were much less likely to move and we were not able to obtain many trials of them running at this temperature.

The low temperature sensitivity observed between 25 and 35°C is likely also, in part, a result of the low muscle activation at 35°C compared with 25°C. At 35°C, hip muscles appear to produce only 58% of their predicted maximum power (Fig. 3). There is little difference in kinematics between 25°C and 35°C, with similar timing of hip, knee and ankle extension, along with a proximal to distal pattern of limb actuation that potentially enables the power amplification observed at both temperatures. However, it is not clear why these lizards do not retain high levels of hip activation and high levels of power amplification at the ankle and the knee in order to achieve higher levels of acceleration and sprint performance at 35°C. The finding that hip muscle activation is decreased, and ankle and knee power amplification are reduced, at 35°C in the absence of a change in kinematics strongly suggests that there is some limit to sprint performance other than the peak instantaneous power that can be generated at the joints by muscle and elastic tissues. It may be that pitching of the body (Williams et al., 2009), a lack of traction (Luo and Stefanyshyn, 2011) or a limit to the total amount of work that can be done on the environment in the time available (Labonte, 2023) instead limit sprint performance at high temperatures. Assuming that this limit exists, it may be that these lizards shift power production from the hip to the ankle where the return of elastic strain energy can contribute to limb power and potentially reduce metabolic cost (Holt and Mayfield, 2023). This redistribution of work and power across the limb has been shown previously (Delabastita et al., 2021; Farris and Sawicki, 2012) and it is known that the tuning of dynamic muscle properties can effectively offload work production from the muscles to the tendons (Biewener, 1998; Gillis and Biewener, 2001; Roberts, 1997). A similar proximal to distal shift as observed here has been seen at the human walk–run transition, presumably to exploit elastic strain energy storage and return in running (Farris and Sawicki, 2012). This apparent reduction in hip muscle activation and in power amplification at the knee and the ankle at 35°C may both contribute to the low *Q*_10_ value of acceleration and sprint performance (Marsh and Bennett, 1986a,b) and suggest that acceleration and sprint performance is limited by some factor other than peak instantaneous muscle power at high temperatures.

Our analysis is constrained by the inherent limitations of inverse dynamics in small animals. Owing to the small size of *S. occidentalis*, we could not use another method to generate more detailed inputs to our analysis (see Astley and Roberts, 2014). However, we consistently used conservative estimates, when necessary, for input variables, including the position of the center of pressure, the in-lever distance and the muscles contributing to joint extension. This conservative approach ensured that our calculated instantaneous muscle powers are, if anything, underestimates of *in vivo* muscle powers. In many vertebrates, energy is transferred from proximal to distal joints via biarticular muscles (Biewener, 2016; Neptune et al., 2004) and co-contraction of agonist or antagonist muscles, which are not directly measurable, may alter muscle powers (Ait-Haddou et al., 2000; Zajac et al., 2002). However, in *Sceloporus* lizards, the ankle extensors are not biarticular as they are in many other tetrapods (Secoy, 1971; our observations), and so proximal to distal energy transfer is unlikely in the ankle of this species. Additionally, when the instantaneous joint powers across the limb are summed, the available muscle across the entire leg is only sufficient to generate a little over half the peak powers we measured (Fig. 4), indicating that there is not enough muscle in the entire limb to power the accelerations observed here. Although we do not discount energy transfer between joints, our treatment of muscle mass means that it cannot be responsible for the high maximum instantaneous *in vivo* muscle powers at joints. In our companion paper (Cobos et al., 2026), we address the potential for muscle–tendon units to store and release energy to generate the powers estimated in the MTUs from this analysis.

### Conclusions

Here, we found evidence of power amplification at the knee and ankle joints in *S. occidentalis* during acceleration, and a lower thermal sensitivity of acceleration and some *in vivo* muscle powers at joints than we would expect based on the presumed thermal sensitivity of muscle. This strongly suggests both that power amplification is important for sprint performance in this species, and that this power amplification contributes to the low thermal sensitivity of locomotor performance between 25 and 35°C. However, we also suggest that effects of behavioral strategy and additional limits to performance can alter apparent thermal sensitivity. Reduced motivation to run at low temperatures appears to contribute to the very high thermal sensitivity of organismal performance between 15 and 25°C. Additionally, a limit to organismal performance other than instantaneous muscle power was observed at 35°C and this, in addition to power amplification, may be responsible for low thermal sensitivity between 25 and 35°C.
